# [1,1′-Bi­cyclo­hexa­ne]-1,1′-diol

**DOI:** 10.1107/S2414314623009690

**Published:** 2023-11-14

**Authors:** Zukisani Mtendeni, Eric Cyriel Hosten, Richard Betz

**Affiliations:** a Nelson Mandela University, Summerstrand Campus, Department of Chemistry, University Way, Summerstrand, PO Box 77000, Port Elizabeth, 6031, South Africa; Goethe-Universität Frankfurt, Germany

**Keywords:** crystal structure

## Abstract

The title compound, C_12_H_22_O_2_, is a symmetric diol derived from the pinacol coupling of cyclo­hexa­none. The asymmetric unit contains three complete mol­ecules. The cyclo­hexane moieties adopt chair conformations. Cooperative hydrogen bonding connects the individual mol­ecules to infinite chains propagating along the crystallographic *a-*axis direction.

## Abstract 2

The title compound is a symmetric diol derived from the pinacol coupling of cyclo­hexa­none. The asymmetric unit contains three complete mol­ecules. Cooperative hydrogen bonding connects the individual mol­ecules into infinite chains propagating along the crystallographic *a*-axis direction.

## Graphical abstract

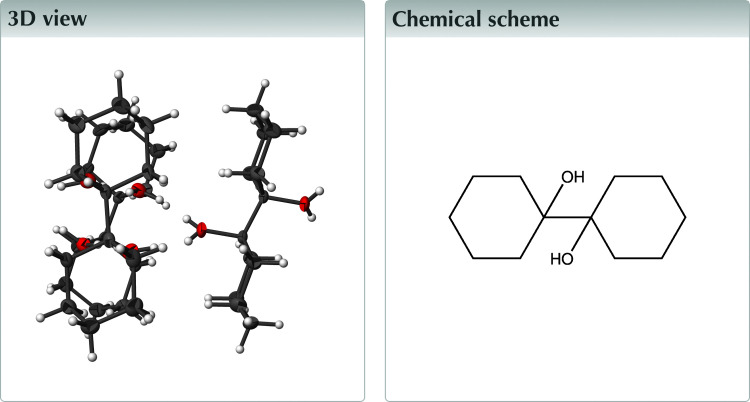

## Structure description

Chelating ligands have found widespread use in coordination chemistry due to the increased stability of coordination compounds they can form in comparison to monodentate ligands (Gade, 1998 ▸). Diols are particularly inter­esting in this aspect as they offer two hydroxyl groups that – depending on the experimental conditions – can either act as fully neutral, fully anionic or mixed neutral-anionic ligands. Upon varying the substitution pattern on the hydro­carbon backbone, the acidity of the respective hydroxyl groups can be fine-tuned over a wide range and they may, thus, serve as probes for establishing the rules in which pK_a_ range coordination to various central atoms can be observed. Furthermore, the steric pretence of potential substituents may give rise to unique coordination and bonding patterns. To allow for comparisons of metrical parameters of the title compound in envisioned coordination compounds, the crystal and mol­ecular structure of the free ligand was determined. The crystal structures of the related pinacols derived from cyclo­penta­none (Hosten & Betz, 2021 ▸), cyclo­hepta­none (Betz & Klüfers, 2007 ▸) and cyclo­dodeca­none (Yang *et al.*, 2016 ▸) are apparent in the literature. Structural data of symmetric pinacols derived from methyl-substituted (Bruss *et al.*, 1987 ▸) and phenyl-substituted cyclo­hexa­nones (Nieger *et al.*, 2004 ▸) have been reported. Furthermore, metrical information based on diffraction studies of other sterically demanding diols such as 1,2-di-*tert*-butyl-ethane-1,2-diol (Kerscher *et al.*, 2009 ▸), 1,2-di­cyclo­pentyl-ethane-1,2-diol (Betz *et al.*, 2007 ▸), as well as *cis*-1,2-dimethyl-cyclo­butane-1,2-diol (Allscher *et al.*, 2008 ▸) are available. The crystal and mol­ecular structure of a coordination compound of osmium featuring the title compound as chelating ligand is apparent in the literature (Lehtonen *et al.*, 1999 ▸).

**Figure 1:**
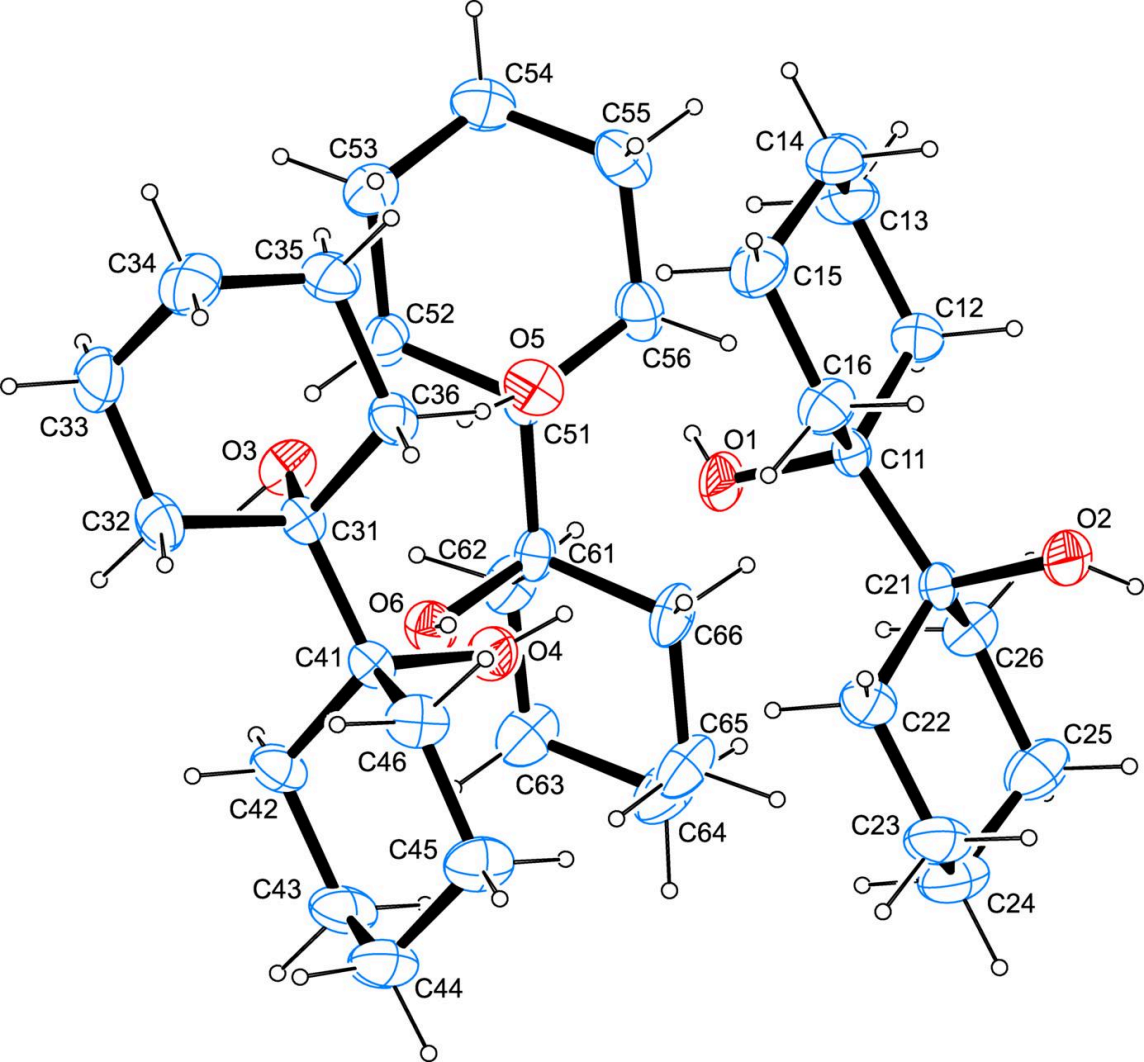
The mol­ecular structure of the title compound, with atom labels and anisotropic displacement ellipsoids (drawn at the 50% probability level). For clarity, only one of the two disordered hydrogen-atom positions is depicted.

The asymmetric unit of the title compound is shown in Fig. 1 ▸ and contains three complete mol­ecules. Bond lengths and angles are normal and in good agreement with values reported for other symmetric pinacols. The hydrogen atoms of the hydroxyl groups are disordered over two orientations. A conformational analysis of the cyclo­hexane rings (Cremer & Pople, 1975 ▸) shows the latter to invariably adopt *chair* conformations (Boeyens, 1978 ▸) with each of the three mol­ecules featuring one ring in a ^1^ *C*_4_ and the second ring in a ^4^ *C*_1_ conformation. In two of the three mol­ecules, the hydroxyl groups adopt a somewhat staggered conformation with respective O—C—C—O torsion angles of 50.95 (11) and 55.14 (10)°, while in the third mol­ecule the two alcoholic groups are arranged in an almost perfect *anti* conformation with the pertaining O—C—C—O angle measuring −177.82 (9)°.

**Figure 2:**
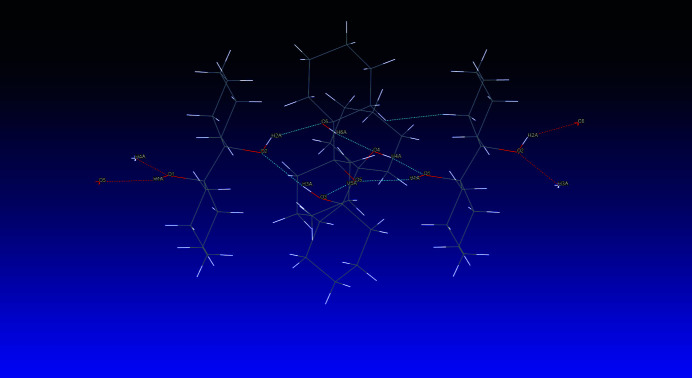
Inter­molecular contacts, viewed approximately along [011]. For clarity, only one of the two disordered hydrogen-atom positions is depicted.

In the crystal, classical hydrogen bonds of the O—H⋯O type are apparent that involve all hydroxyl groups as donors and acceptors (Table 1 ▸), forming cooperative cyclic patterns. In terms of graph-set analysis (Etter *et al.*, 1990 ▸; Bernstein *et al.*, 1995 ▸), the descriptor for these hydrogen bonds is *DDDDDD* on the unary level while an 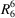 (12) descriptor is required on the hexa­nary level. The mol­ecule featuring the *anti*-orientated hydroxyl groups acts as a linker between these various trimeric arrangements, thus giving rise to infinite chains of the hitherto hydrogen-bonded mol­ecules along the crystallographic *a-*axis direction. A depiction of the pattern is shown in Fig. 2 ▸.

## Synthesis and crystallization

The title compound was synthesized from cyclo­hexa­none according to a published procedure (Criegee *et al.*, 1952 ▸). Crystals suitable for the diffraction study were obtained upon slow evaporation of a solution of the compound in THF at room temperature.

## Refinement

Crystal data, data collection and structure refinement details are summarized in Table 2 ▸. The carbon-bound H atoms of the methyl­ene groups were placed in calculated positions (C–H 0.99 Å) and were included in the refinement in the riding model approximation, with *U*_iso_(H) set to 1.2*U*_eq_(C). The H atoms of the hydroxyl groups were located in a DFM accounting for the equal disorder over two positions, with *U*_iso_(H) set to 1.5*U*_eq_(O).

## Supplementary Material

Crystal structure: contains datablock(s) I, global. DOI: 10.1107/S2414314623009690/bt4141sup1.cif

Structure factors: contains datablock(s) I. DOI: 10.1107/S2414314623009690/bt4141Isup2.hkl

Supporting information file. DOI: 10.1107/S2414314623009690/bt4141Isup3.cml

CCDC reference: 2306283

Additional supporting information:  crystallographic information; 3D view; checkCIF report

## Figures and Tables

**Table 1:** Hydrogen-bond geometry (Å, °)

*D*—H⋯*A*	*D*—H	H⋯*A*	*D*⋯*A*	*D*—H⋯*A*
O1—H1*A*⋯O5	0.78	2.13	2.8182 (12)	149
O1—H1*B*⋯O4	0.77	2.08	2.8179 (11)	161
O2—H2*A*⋯O6^i^	0.81	2.14	2.8315 (11)	143
O2—H2*B*⋯O3^i^	0.71	2.23	2.8551 (12)	147
O3—H3*A*⋯O2^ii^	0.89	2.07	2.8551 (12)	148
O3—H3*B*⋯O5	0.88	1.97	2.8425 (11)	172
O4—H4*A*⋯O1	0.83	2.01	2.8179 (11)	162
O4—H4*B*⋯O5	0.79	2.59	3.1501 (12)	130
O4—H4*B*⋯O6	0.79	2.13	2.8681 (12)	157
O5—H5*A*⋯O3	0.86	1.99	2.8425 (11)	170
O5—H5*B*⋯O1	0.80	2.12	2.8182 (12)	145
O6—H6*A*⋯O4	0.84	2.04	2.8681 (12)	170
O6—H6*A*⋯O5	0.84	2.49	2.7763 (12)	101
O6—H6*B*⋯O2^ii^	0.95	1.91	2.8315 (11)	164
C12—H12*A*⋯O2	0.99	2.57	2.9683 (15)	104
C16—H16*B*⋯O2	0.99	2.49	2.9056 (15)	105
C22—H22*B*⋯O1	0.99	2.51	2.9180 (15)	105
C26—H26*B*⋯O1	0.99	2.57	2.9676 (14)	104
C42—H42*B*⋯O2^ii^	0.99	2.57	3.5090 (15)	159
C52—H52*B*⋯O2^ii^	0.99	2.58	3.5291 (14)	161
C52—H52*B*⋯O6	0.99	2.59	2.9541 (14)	102

Symmetry codes: (i) 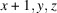 ; (ii) 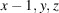 .

**Table 2:** Experimental details

Crystal data	
Chemical formula	C_12_H_22_O_2_
*M*_r_	198.29
Crystal system, space group	Triclinic, *P* 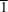
Temperature (K)	200
*a*, *b*, *c* (Å)	9.8996 (7), 10.0299 (7), 17.9841 (13)
α, β, γ (°)	73.810 (3), 86.774 (4), 83.592 (3)
*V* (Å^3^)	1703.6 (2)
*Z*	6
Radiation type	Mo *K*α
μ (mm^−1^)	0.08
Crystal size (mm)	0.60 × 0.49 × 0.18

Data collection	
Diffractometer	Bruker APEXII CCD
Absorption correction	Numerical (*SADABS*; Krause *et al.*, 2015 ▸)
*T*_min_, *T*_max_	0.941, 1.000
No. of measured, independent and observed [*I* > 2σ(*I*)] reflections	30730, 8498, 6202
*R*_int_	0.024
(sin θ/λ)_max_ (Å^−1^)	0.669

Refinement	
*R*[*F* ^2^ > 2σ(*F* ^2^)], *wR*(*F* ^2^), *S*	0.042, 0.110, 1.02
No. of reflections	8498
No. of parameters	380
H-atom treatment	H-atom parameters constrained
Δρ_max_, Δρ_min_ (e Å^−3^)	0.29, −0.19

Computer programs: *APEX2* and *SAINT* (Bruker, 2010 ▸), *SHELXS97* and *SHELXL97* (Sheldrick, 2008 ▸), *PLATON* (Spek, 2020 ▸), *Mercury* (Macrae *et al.*, 2020 ▸) and *SHELXTL* (Sheldrick, 2008 ▸).

## full crystallographic data

### Crystal data

**Table:**

C_12_H_22_O_2_	*Z* = 6
*M**_r_* = 198.29	*F*(000) = 660
Triclinic, *P*1	*D*_x_ = 1.160 Mg m^−^^3^
*a* = 9.8996 (7) Å	Mo *K*α radiation, λ = 0.71073 Å
*b* = 10.0299 (7) Å	Cell parameters from 9559 reflections
*c* = 17.9841 (13) Å	θ = 2.4–28.3°
α = 73.810 (3)°	µ = 0.08 mm^−^^1^
β = 86.774 (4)°	*T* = 200 K
γ = 83.592 (3)°	Blocks, colourless
*V* = 1703.6 (2) Å^3^	0.60 × 0.49 × 0.18 mm

### Data collection

**Table:**

Bruker APEXII CCD diffractometer	8498 independent reflections
Radiation source: sealed tube	6202 reflections with *I* > 2σ(*I*)
Graphite monochromator	*R*_int_ = 0.024
φ and ω scans	θ_max_ = 28.4°, θ_min_ = 2.1°
Absorption correction: numerical (SADABS; Krause *et al.*, 2015)	*h* = −13→13
*T*_min_ = 0.941, *T*_max_ = 1.000	*k* = −13→13
30730 measured reflections	*l* = −23→24

### Refinement

**Table:**

Refinement on *F*^2^	Primary atom site location: structure-invariant direct methods
Least-squares matrix: full	Secondary atom site location: difference Fourier map
*R*[*F*^2^ > 2σ(*F*^2^)] = 0.042	Hydrogen site location: inferred from neighbouring sites
*wR*(*F*^2^) = 0.110	H-atom parameters constrained
*S* = 1.02	*w* = 1/[σ^2^(*F*_o_^2^) + (0.0505*P*)^2^ + 0.3176*P*] where *P* = (*F*_o_^2^ + 2*F*_c_^2^)/3
8498 reflections	(Δ/σ)_max_ = 0.001
380 parameters	Δρ_max_ = 0.29 e Å^−^^3^
0 restraints	Δρ_min_ = −0.19 e Å^−^^3^

### Special details

**Table:**

Geometry. All esds (except the esd in the dihedral angle between two l.s. planes) are estimated using the full covariance matrix. The cell esds are taken into account individually in the estimation of esds in distances, angles and torsion angles; correlations between esds in cell parameters are only used when they are defined by crystal symmetry. An approximate (isotropic) treatment of cell esds is used for estimating esds involving l.s. planes.

### Fractional atomic coordinates and isotropic or equivalent isotropic displacement parameters (Å^2^)

**Table:**

	*x*	*y*	*z*	*U*_iso_*/*U*_eq_	Occ. (<1)
O1	0.56808 (8)	0.29971 (10)	0.76040 (6)	0.0365 (2)	
H1A	0.515794	0.361612	0.741310	0.055*	0.5
H1B	0.523094	0.239516	0.778010	0.055*	0.5
O2	0.93801 (8)	0.22716 (9)	0.73920 (6)	0.0348 (2)	
H2A	0.988346	0.221703	0.703067	0.052*	0.5
H2B	0.963946	0.239903	0.772366	0.052*	0.5
O3	0.14540 (8)	0.28120 (8)	0.82788 (4)	0.02719 (19)	
H3A	0.078291	0.237734	0.817663	0.041*	0.5
H3B	0.198291	0.321434	0.789254	0.027*	0.5
O4	0.36472 (8)	0.11455 (8)	0.79255 (5)	0.02812 (19)	
H4A	0.435244	0.154297	0.788666	0.042*	0.5
H4B	0.332044	0.165297	0.754866	0.042*	0.5
O5	0.31219 (8)	0.43533 (8)	0.70791 (5)	0.02771 (19)	
H5A	0.268910	0.380544	0.744571	0.042*	0.5
H5B	0.393910	0.423744	0.706071	0.042*	0.5
O6	0.19507 (8)	0.23358 (8)	0.66154 (5)	0.02741 (19)	
H6A	0.243395	0.208154	0.700916	0.041*	0.5
H6B	0.101960	0.234254	0.678021	0.041*	0.5
C11	0.70830 (10)	0.32190 (11)	0.76342 (6)	0.0201 (2)	
C12	0.72690 (12)	0.46897 (12)	0.71186 (7)	0.0286 (3)	
H12A	0.825354	0.479829	0.704790	0.034*	
H12B	0.690309	0.479139	0.660247	0.034*	
C13	0.65728 (14)	0.58557 (14)	0.74410 (9)	0.0384 (3)	
H13A	0.679433	0.676950	0.710034	0.046*	
H13B	0.557472	0.583494	0.744701	0.046*	
C14	0.70274 (14)	0.56951 (15)	0.82538 (9)	0.0405 (3)	
H14A	0.655153	0.644961	0.845533	0.049*	
H14B	0.801703	0.577072	0.824556	0.049*	
C15	0.67119 (14)	0.42892 (15)	0.87778 (8)	0.0374 (3)	
H15A	0.571577	0.424348	0.881147	0.045*	
H15B	0.702873	0.418494	0.930530	0.045*	
C16	0.73978 (13)	0.30961 (13)	0.84762 (7)	0.0304 (3)	
H16A	0.710496	0.220570	0.880910	0.037*	
H16B	0.839411	0.306066	0.852129	0.037*	
C21	0.79768 (10)	0.20756 (11)	0.73319 (6)	0.0195 (2)	
C22	0.77309 (13)	0.06019 (12)	0.78313 (7)	0.0305 (3)	
H22A	0.806832	0.047227	0.835712	0.037*	
H22B	0.673976	0.052434	0.788016	0.037*	
C23	0.84177 (14)	−0.05635 (13)	0.75082 (8)	0.0367 (3)	
H23A	0.941711	−0.057487	0.752324	0.044*	
H23B	0.815950	−0.147337	0.783547	0.044*	
C24	0.80068 (15)	−0.03633 (14)	0.66817 (9)	0.0403 (3)	
H24A	0.846954	−0.112472	0.648114	0.048*	
H24B	0.701294	−0.039465	0.666676	0.048*	
C25	0.83963 (14)	0.10308 (15)	0.61805 (8)	0.0374 (3)	
H25A	0.811717	0.116417	0.564250	0.045*	
H25B	0.939634	0.103575	0.617425	0.045*	
C26	0.77213 (12)	0.22312 (13)	0.64813 (7)	0.0273 (3)	
H26A	0.805802	0.311191	0.616424	0.033*	
H26B	0.672844	0.230645	0.641055	0.033*	
C31	0.21725 (11)	0.18418 (11)	0.89152 (6)	0.0200 (2)	
C32	0.11161 (12)	0.14108 (13)	0.95726 (7)	0.0301 (3)	
H32A	0.038665	0.099471	0.938646	0.036*	
H32B	0.155602	0.068901	1.000863	0.036*	
C33	0.04866 (13)	0.26421 (15)	0.98627 (8)	0.0373 (3)	
H33A	−0.004750	0.331300	0.944500	0.045*	
H33B	−0.014316	0.230173	1.030580	0.045*	
C34	0.15628 (14)	0.33828 (16)	1.01140 (8)	0.0386 (3)	
H34A	0.204689	0.274007	1.056236	0.046*	
H34B	0.112317	0.419662	1.027632	0.046*	
C35	0.25723 (13)	0.38674 (13)	0.94504 (8)	0.0325 (3)	
H35A	0.329264	0.430854	0.962745	0.039*	
H35B	0.210061	0.457459	0.902040	0.039*	
C36	0.32149 (11)	0.26445 (12)	0.91615 (7)	0.0256 (2)	
H36A	0.376900	0.199415	0.957738	0.031*	
H36B	0.383335	0.299910	0.871499	0.031*	
C41	0.28844 (11)	0.05912 (11)	0.86257 (6)	0.0204 (2)	
C42	0.18433 (12)	−0.02606 (12)	0.84183 (7)	0.0278 (3)	
H42A	0.126505	−0.062742	0.888178	0.033*	
H42B	0.124784	0.036153	0.801333	0.033*	
C43	0.25017 (14)	−0.14774 (13)	0.81273 (8)	0.0358 (3)	
H43A	0.178583	−0.200925	0.802082	0.043*	
H43B	0.301525	−0.111365	0.763725	0.043*	
C44	0.34571 (15)	−0.24392 (13)	0.87229 (9)	0.0387 (3)	
H44A	0.390810	−0.319501	0.851373	0.046*	
H44B	0.293121	−0.287081	0.919707	0.046*	
C45	0.45267 (14)	−0.16253 (13)	0.89230 (8)	0.0359 (3)	
H45A	0.511192	−0.128125	0.845925	0.043*	
H45B	0.510917	−0.225632	0.933167	0.043*	
C46	0.38887 (13)	−0.03865 (13)	0.92049 (7)	0.0301 (3)	
H46A	0.461886	0.014868	0.929086	0.036*	
H46B	0.340597	−0.073931	0.970704	0.036*	
C51	0.24752 (10)	0.47484 (11)	0.63405 (6)	0.0207 (2)	
C52	0.09989 (11)	0.52910 (12)	0.64770 (7)	0.0245 (2)	
H52A	0.051896	0.555035	0.598091	0.029*	
H52B	0.054060	0.453345	0.684257	0.029*	
C53	0.08875 (13)	0.65513 (13)	0.68017 (7)	0.0301 (3)	
H53A	−0.008295	0.686594	0.687308	0.036*	
H53B	0.131466	0.628397	0.731396	0.036*	
C54	0.15860 (13)	0.77386 (13)	0.62562 (8)	0.0343 (3)	
H54A	0.154109	0.853302	0.648551	0.041*	
H54B	0.111170	0.806044	0.575789	0.041*	
C55	0.30656 (13)	0.72491 (13)	0.61147 (8)	0.0353 (3)	
H55A	0.355879	0.704091	0.660420	0.042*	
H55B	0.348864	0.801160	0.573165	0.042*	
C56	0.32183 (12)	0.59495 (13)	0.58173 (7)	0.0294 (3)	
H56A	0.419645	0.562844	0.578249	0.035*	
H56B	0.285412	0.619887	0.528935	0.035*	
C61	0.25485 (10)	0.34261 (12)	0.60330 (6)	0.0209 (2)	
C62	0.17544 (12)	0.36547 (13)	0.52915 (7)	0.0280 (3)	
H62A	0.078056	0.390171	0.539822	0.034*	
H62B	0.208641	0.444956	0.488752	0.034*	
C63	0.18968 (13)	0.23674 (15)	0.49851 (8)	0.0352 (3)	
H63A	0.144320	0.161208	0.535547	0.042*	
H63B	0.143072	0.259795	0.448569	0.042*	
C64	0.33798 (14)	0.18484 (16)	0.48677 (8)	0.0391 (3)	
H64A	0.342635	0.098089	0.470299	0.047*	
H64B	0.381297	0.255816	0.445447	0.047*	
C65	0.41386 (13)	0.15677 (15)	0.56150 (8)	0.0353 (3)	
H65A	0.510863	0.127499	0.552576	0.042*	
H65B	0.375663	0.079819	0.601439	0.042*	
C66	0.40235 (11)	0.28693 (14)	0.59025 (7)	0.0283 (3)	
H66A	0.447745	0.360803	0.551963	0.034*	
H66B	0.450876	0.265098	0.639524	0.034*	

### Atomic displacement parameters (Å^2^)

**Table:**

	*U*^11^	*U*^22^	*U*^33^	*U*^12^	*U*^13^	*U*^23^
O1	0.0136 (4)	0.0469 (6)	0.0588 (6)	−0.0060 (4)	0.0016 (4)	−0.0298 (5)
O2	0.0141 (4)	0.0387 (5)	0.0583 (6)	−0.0014 (3)	−0.0053 (4)	−0.0238 (4)
O3	0.0292 (4)	0.0283 (4)	0.0214 (4)	0.0039 (3)	−0.0054 (3)	−0.0041 (3)
O4	0.0303 (4)	0.0261 (4)	0.0248 (4)	−0.0019 (3)	0.0046 (3)	−0.0031 (3)
O5	0.0307 (4)	0.0289 (4)	0.0224 (4)	−0.0032 (3)	−0.0099 (3)	−0.0032 (3)
O6	0.0322 (4)	0.0269 (4)	0.0224 (4)	−0.0062 (3)	0.0028 (3)	−0.0049 (3)
C11	0.0134 (5)	0.0236 (5)	0.0237 (5)	−0.0030 (4)	−0.0018 (4)	−0.0068 (4)
C12	0.0285 (6)	0.0220 (6)	0.0339 (6)	−0.0021 (5)	0.0011 (5)	−0.0060 (5)
C13	0.0366 (7)	0.0257 (7)	0.0540 (9)	0.0016 (5)	−0.0029 (6)	−0.0142 (6)
C14	0.0332 (7)	0.0404 (8)	0.0595 (9)	−0.0046 (6)	−0.0007 (6)	−0.0323 (7)
C15	0.0332 (7)	0.0497 (8)	0.0378 (7)	−0.0039 (6)	0.0011 (6)	−0.0266 (6)
C16	0.0319 (6)	0.0357 (7)	0.0258 (6)	−0.0004 (5)	−0.0023 (5)	−0.0127 (5)
C21	0.0133 (5)	0.0227 (5)	0.0230 (5)	−0.0023 (4)	−0.0023 (4)	−0.0066 (4)
C22	0.0346 (6)	0.0224 (6)	0.0320 (6)	−0.0028 (5)	0.0004 (5)	−0.0037 (5)
C23	0.0400 (7)	0.0225 (6)	0.0479 (8)	0.0022 (5)	−0.0084 (6)	−0.0107 (6)
C24	0.0390 (7)	0.0350 (7)	0.0544 (9)	0.0052 (6)	−0.0131 (6)	−0.0258 (7)
C25	0.0381 (7)	0.0459 (8)	0.0327 (7)	0.0054 (6)	−0.0043 (6)	−0.0211 (6)
C26	0.0273 (6)	0.0307 (6)	0.0245 (6)	0.0022 (5)	−0.0043 (5)	−0.0096 (5)
C31	0.0200 (5)	0.0220 (5)	0.0174 (5)	−0.0045 (4)	−0.0033 (4)	−0.0030 (4)
C32	0.0308 (6)	0.0334 (7)	0.0259 (6)	−0.0116 (5)	0.0050 (5)	−0.0055 (5)
C33	0.0333 (7)	0.0467 (8)	0.0339 (7)	−0.0088 (6)	0.0116 (5)	−0.0149 (6)
C34	0.0420 (7)	0.0470 (8)	0.0325 (7)	−0.0024 (6)	0.0015 (6)	−0.0215 (6)
C35	0.0337 (6)	0.0318 (7)	0.0379 (7)	−0.0071 (5)	−0.0035 (5)	−0.0172 (5)
C36	0.0231 (5)	0.0305 (6)	0.0263 (6)	−0.0070 (5)	−0.0026 (4)	−0.0110 (5)
C41	0.0212 (5)	0.0202 (5)	0.0191 (5)	−0.0043 (4)	−0.0039 (4)	−0.0025 (4)
C42	0.0269 (6)	0.0230 (6)	0.0344 (6)	−0.0051 (5)	−0.0087 (5)	−0.0069 (5)
C43	0.0387 (7)	0.0278 (6)	0.0465 (8)	−0.0048 (5)	−0.0130 (6)	−0.0164 (6)
C44	0.0456 (8)	0.0222 (6)	0.0478 (8)	0.0008 (6)	−0.0074 (6)	−0.0091 (6)
C45	0.0379 (7)	0.0282 (7)	0.0399 (7)	0.0069 (5)	−0.0160 (6)	−0.0077 (6)
C46	0.0344 (6)	0.0276 (6)	0.0279 (6)	0.0010 (5)	−0.0127 (5)	−0.0061 (5)
C51	0.0169 (5)	0.0250 (6)	0.0178 (5)	−0.0011 (4)	−0.0032 (4)	−0.0016 (4)
C52	0.0184 (5)	0.0263 (6)	0.0276 (6)	0.0000 (4)	−0.0014 (4)	−0.0060 (5)
C53	0.0290 (6)	0.0286 (6)	0.0325 (6)	0.0009 (5)	−0.0008 (5)	−0.0094 (5)
C54	0.0400 (7)	0.0252 (6)	0.0362 (7)	−0.0019 (5)	−0.0056 (6)	−0.0060 (5)
C55	0.0360 (7)	0.0294 (7)	0.0380 (7)	−0.0108 (5)	−0.0008 (6)	−0.0025 (5)
C56	0.0265 (6)	0.0310 (6)	0.0265 (6)	−0.0056 (5)	0.0023 (5)	−0.0003 (5)
C61	0.0156 (5)	0.0271 (6)	0.0182 (5)	−0.0001 (4)	−0.0011 (4)	−0.0038 (4)
C62	0.0237 (5)	0.0370 (7)	0.0232 (6)	0.0034 (5)	−0.0064 (4)	−0.0094 (5)
C63	0.0309 (6)	0.0469 (8)	0.0321 (7)	0.0050 (6)	−0.0098 (5)	−0.0196 (6)
C64	0.0362 (7)	0.0531 (9)	0.0330 (7)	0.0083 (6)	−0.0047 (6)	−0.0246 (6)
C65	0.0266 (6)	0.0467 (8)	0.0343 (7)	0.0107 (6)	−0.0036 (5)	−0.0189 (6)
C66	0.0171 (5)	0.0435 (7)	0.0247 (6)	0.0029 (5)	−0.0018 (4)	−0.0119 (5)

### Geometric parameters (Å, º)

**Table:**

O1—C11	1.4366 (12)	C33—H33B	0.9900
O1—H1A	0.7750	C34—C35	1.5199 (18)
O1—H1B	0.7750	C34—H34A	0.9900
O2—C21	1.4387 (12)	C34—H34B	0.9900
O2—H2A	0.8069	C35—C36	1.5242 (17)
O2—H2B	0.7106	C35—H35A	0.9900
O3—C31	1.4418 (13)	C35—H35B	0.9900
O3—H3A	0.8879	C36—H36A	0.9900
O3—H3B	0.8772	C36—H36B	0.9900
O4—C41	1.4357 (12)	C41—C42	1.5325 (15)
O4—H4A	0.8321	C41—C46	1.5377 (16)
O4—H4B	0.7865	C42—C43	1.5259 (18)
O5—C51	1.4412 (13)	C42—H42A	0.9900
O5—H5A	0.8596	C42—H42B	0.9900
O5—H5B	0.8045	C43—C44	1.5187 (19)
O6—C61	1.4419 (12)	C43—H43A	0.9900
O6—H6A	0.8399	C43—H43B	0.9900
O6—H6B	0.9521	C44—C45	1.5220 (19)
C11—C16	1.5317 (16)	C44—H44A	0.9900
C11—C12	1.5324 (15)	C44—H44B	0.9900
C11—C21	1.5685 (15)	C45—C46	1.5296 (18)
C12—C13	1.5270 (18)	C45—H45A	0.9900
C12—H12A	0.9900	C45—H45B	0.9900
C12—H12B	0.9900	C46—H46A	0.9900
C13—C14	1.514 (2)	C46—H46B	0.9900
C13—H13A	0.9900	C51—C52	1.5326 (15)
C13—H13B	0.9900	C51—C56	1.5380 (15)
C14—C15	1.516 (2)	C51—C61	1.5662 (16)
C14—H14A	0.9900	C52—C53	1.5246 (17)
C14—H14B	0.9900	C52—H52A	0.9900
C15—C16	1.5252 (18)	C52—H52B	0.9900
C15—H15A	0.9900	C53—C54	1.5214 (17)
C15—H15B	0.9900	C53—H53A	0.9900
C16—H16A	0.9900	C53—H53B	0.9900
C16—H16B	0.9900	C54—C55	1.5226 (18)
C21—C26	1.5265 (16)	C54—H54A	0.9900
C21—C22	1.5382 (15)	C54—H54B	0.9900
C22—C23	1.5241 (18)	C55—C56	1.5309 (18)
C22—H22A	0.9900	C55—H55A	0.9900
C22—H22B	0.9900	C55—H55B	0.9900
C23—C24	1.516 (2)	C56—H56A	0.9900
C23—H23A	0.9900	C56—H56B	0.9900
C23—H23B	0.9900	C61—C66	1.5335 (15)
C24—C25	1.515 (2)	C61—C62	1.5350 (16)
C24—H24A	0.9900	C62—C63	1.5290 (18)
C24—H24B	0.9900	C62—H62A	0.9900
C25—C26	1.5270 (18)	C62—H62B	0.9900
C25—H25A	0.9900	C63—C64	1.5254 (18)
C25—H25B	0.9900	C63—H63A	0.9900
C26—H26A	0.9900	C63—H63B	0.9900
C26—H26B	0.9900	C64—C65	1.5183 (18)
C31—C36	1.5314 (15)	C64—H64A	0.9900
C31—C32	1.5346 (14)	C64—H64B	0.9900
C31—C41	1.5705 (16)	C65—C66	1.5236 (18)
C32—C33	1.5287 (18)	C65—H65A	0.9900
C32—H32A	0.9900	C65—H65B	0.9900
C32—H32B	0.9900	C66—H66A	0.9900
C33—C34	1.5196 (19)	C66—H66B	0.9900
C33—H33A	0.9900		
			
C11—O1—H1A	120.0	C34—C35—H35B	109.5
C11—O1—H1B	136.4	C36—C35—H35B	109.5
H1A—O1—H1B	103.2	H35A—C35—H35B	108.1
C21—O2—H2A	117.1	C35—C36—C31	113.42 (10)
C21—O2—H2B	123.0	C35—C36—H36A	108.9
H2A—O2—H2B	119.9	C31—C36—H36A	108.9
C31—O3—H3A	106.4	C35—C36—H36B	108.9
C31—O3—H3B	114.1	C31—C36—H36B	108.9
H3A—O3—H3B	118.1	H36A—C36—H36B	107.7
C41—O4—H4A	126.5	O4—C41—C42	106.37 (9)
C41—O4—H4B	123.6	O4—C41—C46	107.05 (9)
H4A—O4—H4B	95.3	C42—C41—C46	109.45 (9)
C51—O5—H5A	114.5	O4—C41—C31	108.51 (8)
C51—O5—H5B	115.3	C42—C41—C31	111.61 (9)
H5A—O5—H5B	118.9	C46—C41—C31	113.48 (9)
C61—O6—H6A	109.8	C43—C42—C41	112.97 (10)
C61—O6—H6B	126.1	C43—C42—H42A	109.0
H6A—O6—H6B	108.6	C41—C42—H42A	109.0
O1—C11—C16	108.21 (9)	C43—C42—H42B	109.0
O1—C11—C12	107.43 (9)	C41—C42—H42B	109.0
C16—C11—C12	110.59 (10)	H42A—C42—H42B	107.8
O1—C11—C21	108.01 (9)	C44—C43—C42	110.78 (11)
C16—C11—C21	110.92 (9)	C44—C43—H43A	109.5
C12—C11—C21	111.52 (8)	C42—C43—H43A	109.5
C13—C12—C11	113.94 (10)	C44—C43—H43B	109.5
C13—C12—H12A	108.8	C42—C43—H43B	109.5
C11—C12—H12A	108.8	H43A—C43—H43B	108.1
C13—C12—H12B	108.8	C43—C44—C45	110.29 (11)
C11—C12—H12B	108.8	C43—C44—H44A	109.6
H12A—C12—H12B	107.7	C45—C44—H44A	109.6
C14—C13—C12	111.01 (11)	C43—C44—H44B	109.6
C14—C13—H13A	109.4	C45—C44—H44B	109.6
C12—C13—H13A	109.4	H44A—C44—H44B	108.1
C14—C13—H13B	109.4	C44—C45—C46	112.07 (11)
C12—C13—H13B	109.4	C44—C45—H45A	109.2
H13A—C13—H13B	108.0	C46—C45—H45A	109.2
C13—C14—C15	109.87 (11)	C44—C45—H45B	109.2
C13—C14—H14A	109.7	C46—C45—H45B	109.2
C15—C14—H14A	109.7	H45A—C45—H45B	107.9
C13—C14—H14B	109.7	C45—C46—C41	112.44 (10)
C15—C14—H14B	109.7	C45—C46—H46A	109.1
H14A—C14—H14B	108.2	C41—C46—H46A	109.1
C14—C15—C16	111.40 (11)	C45—C46—H46B	109.1
C14—C15—H15A	109.3	C41—C46—H46B	109.1
C16—C15—H15A	109.3	H46A—C46—H46B	107.8
C14—C15—H15B	109.3	O5—C51—C52	107.05 (9)
C16—C15—H15B	109.3	O5—C51—C56	106.55 (9)
H15A—C15—H15B	108.0	C52—C51—C56	109.20 (9)
C15—C16—C11	114.03 (11)	O5—C51—C61	108.20 (8)
C15—C16—H16A	108.7	C52—C51—C61	111.28 (9)
C11—C16—H16A	108.7	C56—C51—C61	114.21 (9)
C15—C16—H16B	108.7	C53—C52—C51	112.79 (10)
C11—C16—H16B	108.7	C53—C52—H52A	109.0
H16A—C16—H16B	107.6	C51—C52—H52A	109.0
O2—C21—C26	107.70 (9)	C53—C52—H52B	109.0
O2—C21—C22	108.24 (9)	C51—C52—H52B	109.0
C26—C21—C22	110.64 (10)	H52A—C52—H52B	107.8
O2—C21—C11	107.67 (8)	C54—C53—C52	110.60 (10)
C26—C21—C11	111.40 (9)	C54—C53—H53A	109.5
C22—C21—C11	111.03 (9)	C52—C53—H53A	109.5
C23—C22—C21	113.89 (10)	C54—C53—H53B	109.5
C23—C22—H22A	108.8	C52—C53—H53B	109.5
C21—C22—H22A	108.8	H53A—C53—H53B	108.1
C23—C22—H22B	108.8	C53—C54—C55	110.03 (10)
C21—C22—H22B	108.8	C53—C54—H54A	109.7
H22A—C22—H22B	107.7	C55—C54—H54A	109.7
C24—C23—C22	111.02 (11)	C53—C54—H54B	109.7
C24—C23—H23A	109.4	C55—C54—H54B	109.7
C22—C23—H23A	109.4	H54A—C54—H54B	108.2
C24—C23—H23B	109.4	C54—C55—C56	112.60 (11)
C22—C23—H23B	109.4	C54—C55—H55A	109.1
H23A—C23—H23B	108.0	C56—C55—H55A	109.1
C25—C24—C23	109.52 (11)	C54—C55—H55B	109.1
C25—C24—H24A	109.8	C56—C55—H55B	109.1
C23—C24—H24A	109.8	H55A—C55—H55B	107.8
C25—C24—H24B	109.8	C55—C56—C51	112.75 (10)
C23—C24—H24B	109.8	C55—C56—H56A	109.0
H24A—C24—H24B	108.2	C51—C56—H56A	109.0
C24—C25—C26	111.29 (11)	C55—C56—H56B	109.0
C24—C25—H25A	109.4	C51—C56—H56B	109.0
C26—C25—H25A	109.4	H56A—C56—H56B	107.8
C24—C25—H25B	109.4	O6—C61—C66	106.78 (9)
C26—C25—H25B	109.4	O6—C61—C62	106.53 (9)
H25A—C25—H25B	108.0	C66—C61—C62	109.43 (9)
C21—C26—C25	114.23 (10)	O6—C61—C51	108.35 (8)
C21—C26—H26A	108.7	C66—C61—C51	111.55 (9)
C25—C26—H26A	108.7	C62—C61—C51	113.83 (9)
C21—C26—H26B	108.7	C63—C62—C61	112.64 (10)
C25—C26—H26B	108.7	C63—C62—H62A	109.1
H26A—C26—H26B	107.6	C61—C62—H62A	109.1
O3—C31—C36	106.58 (9)	C63—C62—H62B	109.1
O3—C31—C32	106.55 (9)	C61—C62—H62B	109.1
C36—C31—C32	109.71 (9)	H62A—C62—H62B	107.8
O3—C31—C41	108.42 (8)	C64—C63—C62	112.29 (11)
C36—C31—C41	111.44 (9)	C64—C63—H63A	109.1
C32—C31—C41	113.76 (9)	C62—C63—H63A	109.1
C33—C32—C31	112.37 (10)	C64—C63—H63B	109.1
C33—C32—H32A	109.1	C62—C63—H63B	109.1
C31—C32—H32A	109.1	H63A—C63—H63B	107.9
C33—C32—H32B	109.1	C65—C64—C63	110.19 (10)
C31—C32—H32B	109.1	C65—C64—H64A	109.6
H32A—C32—H32B	107.9	C63—C64—H64A	109.6
C34—C33—C32	111.83 (11)	C65—C64—H64B	109.6
C34—C33—H33A	109.3	C63—C64—H64B	109.6
C32—C33—H33A	109.3	H64A—C64—H64B	108.1
C34—C33—H33B	109.3	C64—C65—C66	110.68 (11)
C32—C33—H33B	109.3	C64—C65—H65A	109.5
H33A—C33—H33B	107.9	C66—C65—H65A	109.5
C33—C34—C35	110.14 (11)	C64—C65—H65B	109.5
C33—C34—H34A	109.6	C66—C65—H65B	109.5
C35—C34—H34A	109.6	H65A—C65—H65B	108.1
C33—C34—H34B	109.6	C65—C66—C61	113.16 (10)
C35—C34—H34B	109.6	C65—C66—H66A	108.9
H34A—C34—H34B	108.1	C61—C66—H66A	108.9
C34—C35—C36	110.82 (10)	C65—C66—H66B	108.9
C34—C35—H35A	109.5	C61—C66—H66B	108.9
C36—C35—H35A	109.5	H66A—C66—H66B	107.8
			
O1—C11—C12—C13	69.48 (13)	C32—C31—C41—C42	52.39 (12)
C16—C11—C12—C13	−48.41 (13)	O3—C31—C41—C46	169.81 (9)
C21—C11—C12—C13	−172.34 (10)	C36—C31—C41—C46	52.81 (12)
C11—C12—C13—C14	54.76 (14)	C32—C31—C41—C46	−71.85 (12)
C12—C13—C14—C15	−58.26 (14)	O4—C41—C42—C43	61.27 (12)
C13—C14—C15—C16	57.90 (15)	C46—C41—C42—C43	−54.06 (13)
C14—C15—C16—C11	−53.81 (14)	C31—C41—C42—C43	179.46 (9)
O1—C11—C16—C15	−69.52 (13)	C41—C42—C43—C44	57.10 (14)
C12—C11—C16—C15	47.90 (13)	C42—C43—C44—C45	−56.49 (15)
C21—C11—C16—C15	172.17 (9)	C43—C44—C45—C46	55.87 (15)
O1—C11—C21—O2	−177.82 (9)	C44—C45—C46—C41	−54.71 (14)
C16—C11—C21—O2	−59.39 (11)	O4—C41—C46—C45	−62.50 (12)
C12—C11—C21—O2	64.35 (11)	C42—C41—C46—C45	52.40 (13)
O1—C11—C21—C26	64.30 (11)	C31—C41—C46—C45	177.81 (9)
C16—C11—C21—C26	−177.27 (9)	O5—C51—C52—C53	60.10 (11)
C12—C11—C21—C26	−53.53 (12)	C56—C51—C52—C53	−54.88 (12)
O1—C11—C21—C22	−59.50 (12)	C61—C51—C52—C53	178.14 (9)
C16—C11—C21—C22	58.93 (12)	C51—C52—C53—C54	58.53 (13)
C12—C11—C21—C22	−177.33 (10)	C52—C53—C54—C55	−56.80 (14)
O2—C21—C22—C23	−69.88 (13)	C53—C54—C55—C56	54.94 (14)
C26—C21—C22—C23	47.91 (14)	C54—C55—C56—C51	−53.52 (14)
C11—C21—C22—C23	172.14 (10)	O5—C51—C56—C55	−63.46 (12)
C21—C22—C23—C24	−54.81 (15)	C52—C51—C56—C55	51.84 (13)
C22—C23—C24—C25	58.89 (15)	C61—C51—C56—C55	177.14 (10)
C23—C24—C25—C26	−58.37 (15)	O5—C51—C61—O6	55.14 (10)
O2—C21—C26—C25	70.69 (12)	C52—C51—C61—O6	−62.20 (11)
C22—C21—C26—C25	−47.43 (13)	C56—C51—C61—O6	173.61 (9)
C11—C21—C26—C25	−171.45 (10)	O5—C51—C61—C66	−62.12 (10)
C24—C25—C26—C21	53.99 (14)	C52—C51—C61—C66	−179.45 (9)
O3—C31—C32—C33	−62.96 (13)	C56—C51—C61—C66	56.35 (12)
C36—C31—C32—C33	52.04 (13)	O5—C51—C61—C62	173.45 (8)
C41—C31—C32—C33	177.62 (10)	C52—C51—C61—C62	56.11 (11)
C31—C32—C33—C34	−55.49 (15)	C56—C51—C61—C62	−68.09 (12)
C32—C33—C34—C35	56.84 (15)	O6—C61—C62—C63	−63.23 (12)
C33—C34—C35—C36	−56.50 (15)	C66—C61—C62—C63	51.86 (13)
C34—C35—C36—C31	56.00 (14)	C51—C61—C62—C63	177.42 (9)
O3—C31—C36—C35	62.11 (12)	C61—C62—C63—C64	−54.15 (14)
C32—C31—C36—C35	−52.88 (13)	C62—C63—C64—C65	55.53 (16)
C41—C31—C36—C35	−179.78 (9)	C63—C64—C65—C66	−56.49 (15)
O3—C31—C41—O4	50.95 (11)	C64—C65—C66—C61	57.40 (14)
C36—C31—C41—O4	−66.05 (11)	O6—C61—C66—C65	60.90 (12)
C32—C31—C41—O4	169.29 (9)	C62—C61—C66—C65	−54.03 (13)
O3—C31—C41—C42	−65.95 (11)	C51—C61—C66—C65	179.10 (9)
C36—C31—C41—C42	177.05 (9)		

### Hydrogen-bond geometry (Å, º)

**Table:**

*D*—H···*A*	*D*—H	H···*A*	*D*···*A*	*D*—H···*A*
O1—H1*A*···O5	0.78	2.13	2.8182 (12)	149
O1—H1*B*···O4	0.77	2.08	2.8179 (11)	161
O2—H2*A*···O6^i^	0.81	2.14	2.8315 (11)	143
O2—H2*B*···O3^i^	0.71	2.23	2.8551 (12)	147
O3—H3*A*···O2^ii^	0.89	2.07	2.8551 (12)	148
O3—H3*B*···O5	0.88	1.97	2.8425 (11)	172
O4—H4*A*···O1	0.83	2.01	2.8179 (11)	162
O4—H4*B*···O5	0.79	2.59	3.1501 (12)	130
O4—H4*B*···O6	0.79	2.13	2.8681 (12)	157
O5—H5*A*···O3	0.86	1.99	2.8425 (11)	170
O5—H5*B*···O1	0.80	2.12	2.8182 (12)	145
O6—H6*A*···O4	0.84	2.04	2.8681 (12)	170
O6—H6*A*···O5	0.84	2.49	2.7763 (12)	101
O6—H6*B*···O2^ii^	0.95	1.91	2.8315 (11)	164
C12—H12*A*···O2	0.99	2.57	2.9683 (15)	104
C16—H16*B*···O2	0.99	2.49	2.9056 (15)	105
C22—H22*B*···O1	0.99	2.51	2.9180 (15)	105
C26—H26*B*···O1	0.99	2.57	2.9676 (14)	104
C42—H42*B*···O2^ii^	0.99	2.57	3.5090 (15)	159
C52—H52*B*···O2^ii^	0.99	2.58	3.5291 (14)	161
C52—H52*B*···O6	0.99	2.59	2.9541 (14)	102

Symmetry codes: (i) *x*+1, *y*, *z*; (ii) *x*−1, *y*, *z*.
